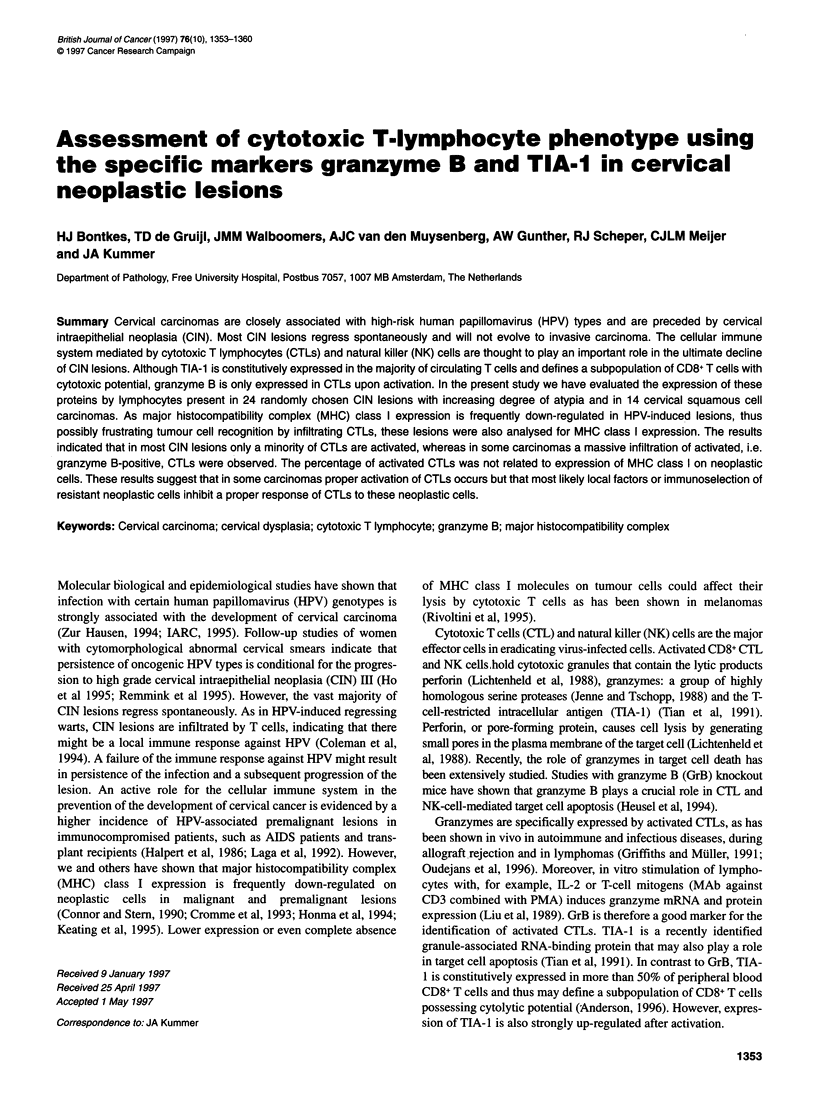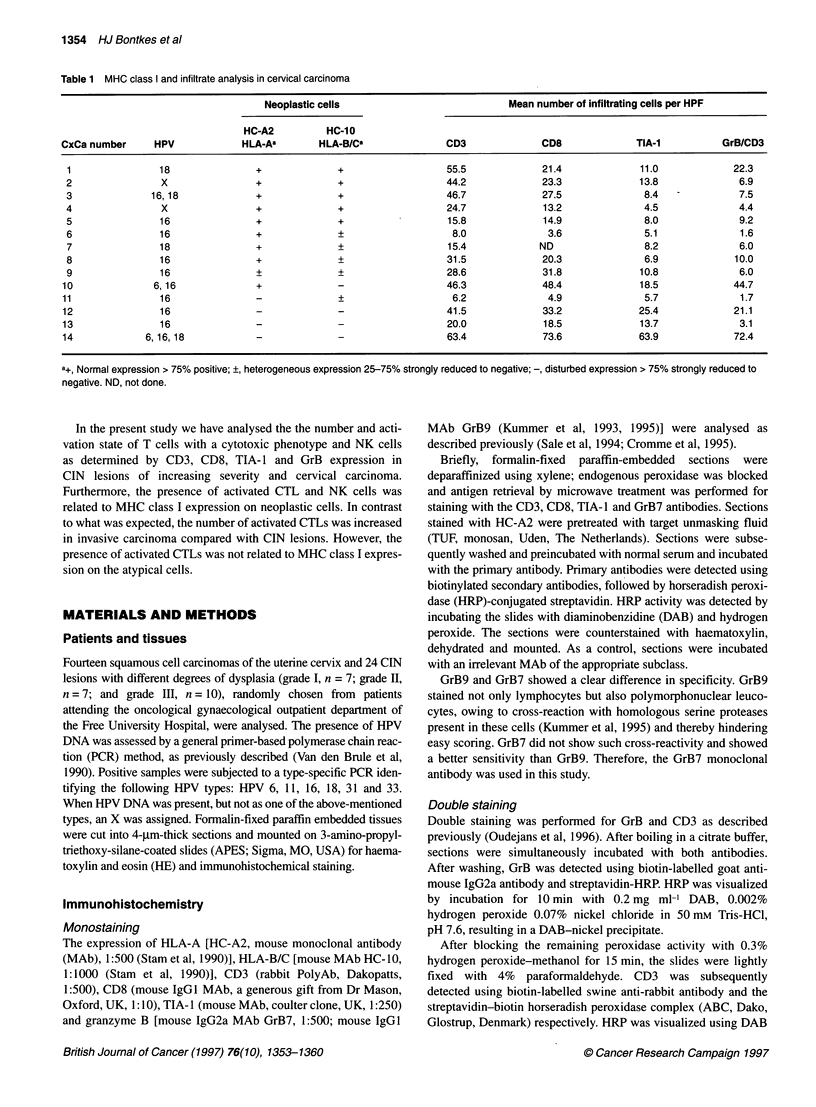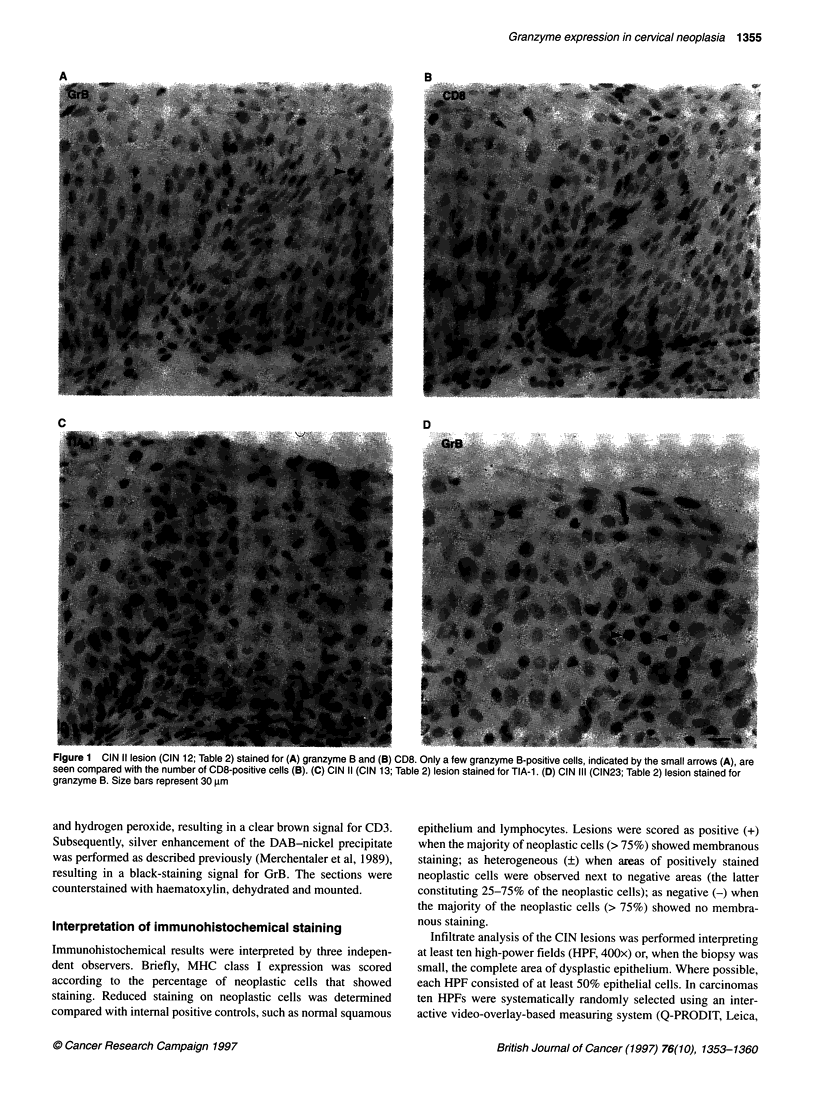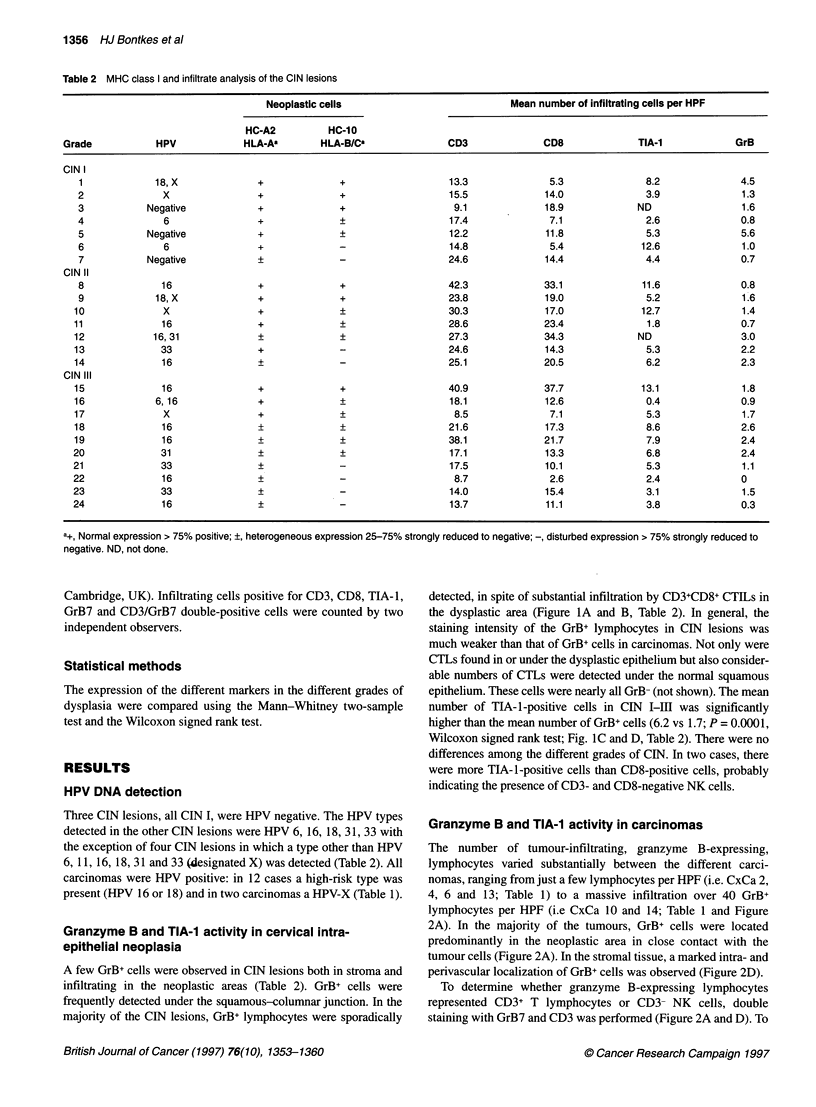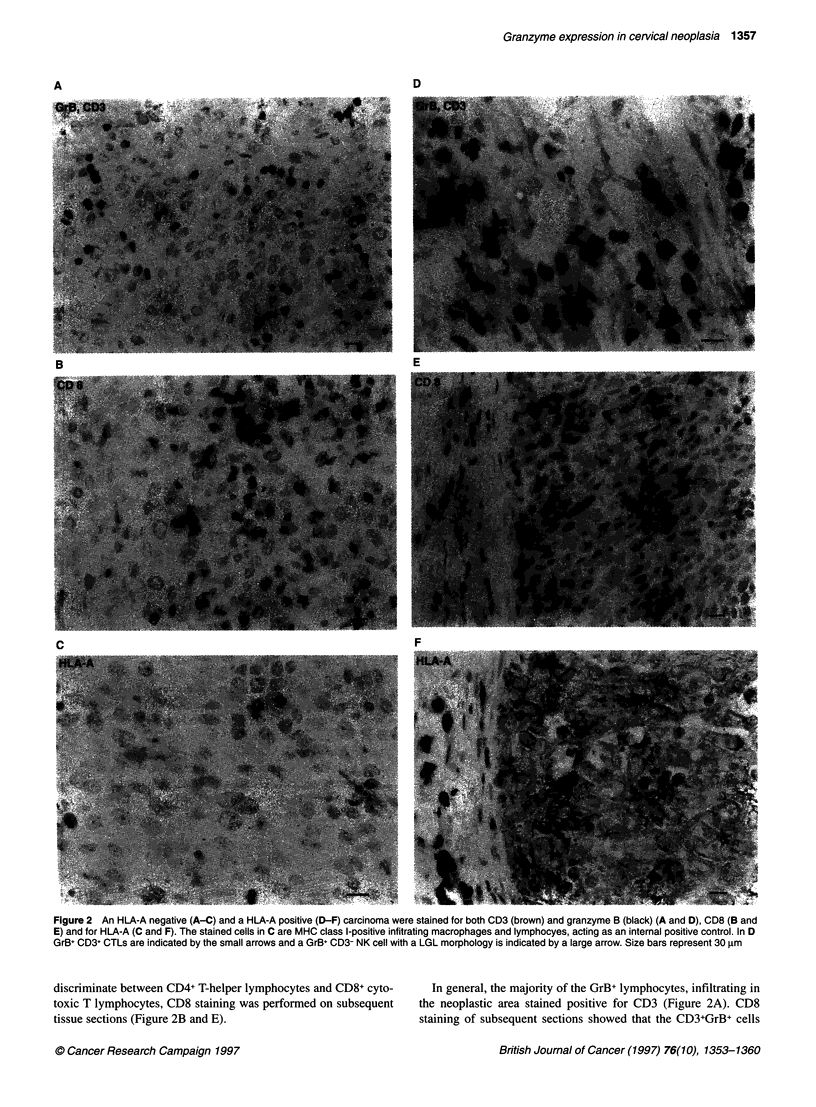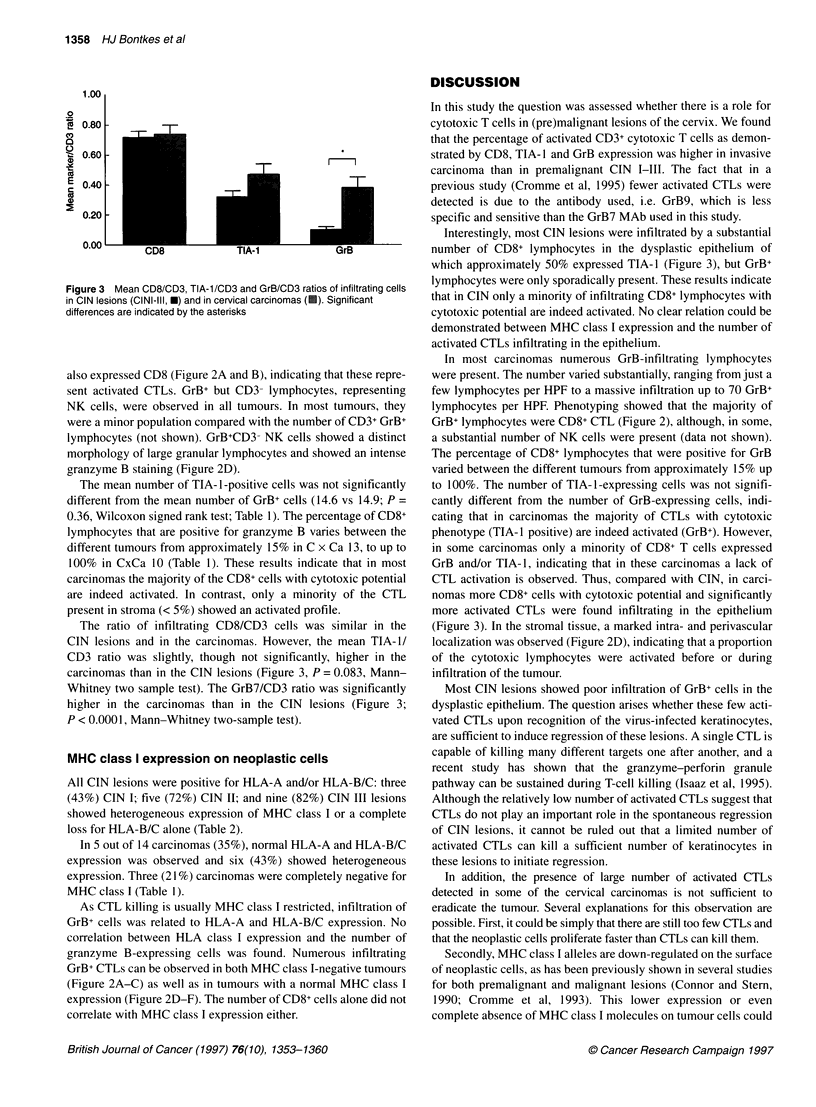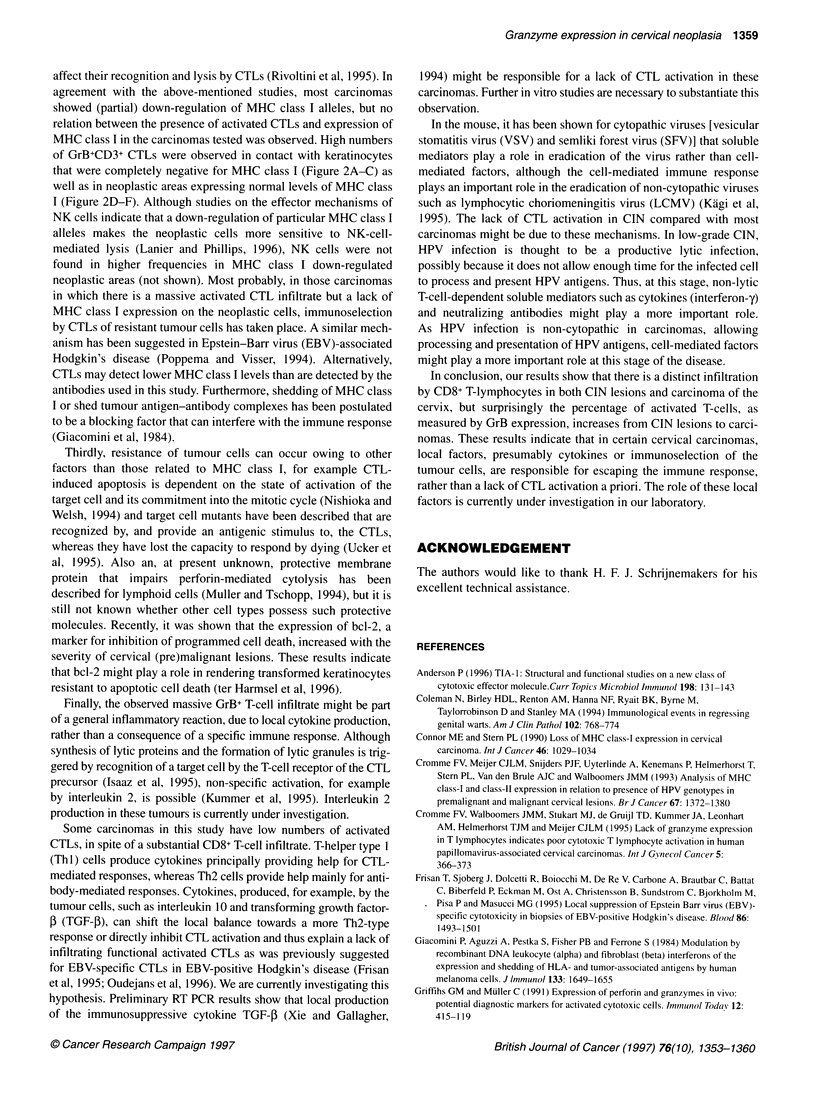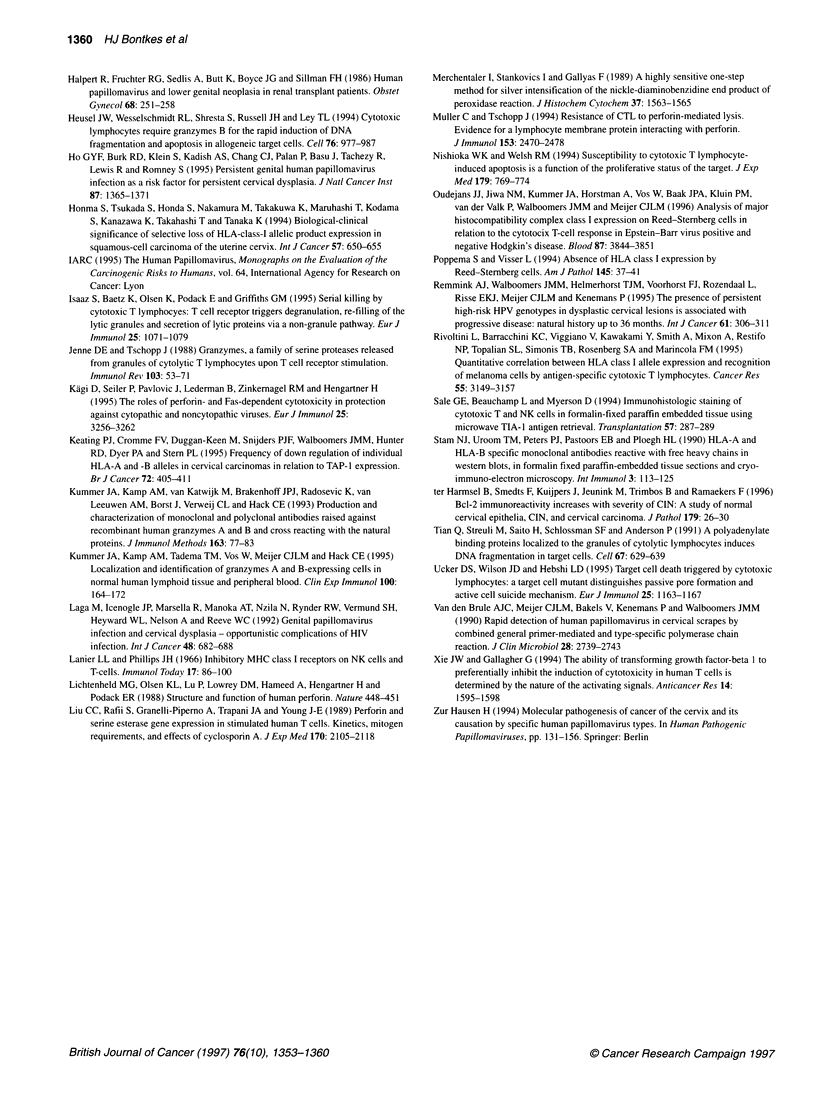# Assessment of cytotoxic T-lymphocyte phenotype using the specific markers granzyme B and TIA-1 in cervical neoplastic lesions.

**DOI:** 10.1038/bjc.1997.560

**Published:** 1997

**Authors:** H. J. Bontkes, T. D. de Gruijl, J. M. Walboomers, A. J. van den Muysenberg, A. W. Gunther, R. J. Scheper, C. J. Meijer, J. A. Kummer

**Affiliations:** Department of Pathology, Free University Hospital, Amsterdam, The Netherlands.

## Abstract

**Images:**


British Journal of Cancer (1997) 76(10), 1353–1360
© 1997 Cancer Research Campaign

### Assessment of cytotoxic T-lymphocyte phenotype using the specific markers granzyme B and TIA-1 in cervical neoplastic lesions

**HJ Bontkes, TD de Gruijl, JMM Walboomers, AJC van den Muysenberg, AW Gunther, RJ Scheper, CJLM Meijer and JA Kummer**

Department of Pathology, Free University Hospital, Postbus 7057, 1007 MB Amsterdam, The Netherlands

**Summary** Cervical carcinomas are closely associated with high-risk human papillomavirus (HPV) types and are preceded by cervical intraepithelial neoplasia (CIN). Most CIN lesions regress spontaneously and will not evolve to invasive carcinoma. The cellular immune system mediated by cytotoxic T lymphocytes (CTLs) and natural killer (NK) cells are thought to play an important role in the ultimate decline of CIN lesions. Although TIA-1 is constitutively expressed in the majority of circulating T cells and defines a subpopulation of $CD8^+$ T cells with cytotoxic potential, granzyme B is only expressed in CTLs upon activation. In the present study we have evaluated the expression of these proteins by lymphocytes present in 24 randomly chosen CIN lesions with increasing degree of atypia and in 14 cervical squamous cell carcinomas. As major histocompatibility complex (MHC) class I expression is frequently down-regulated in HPV-induced lesions, thus possibly frustrating tumour cell recognition by infiltrating CTLs, these lesions were also analysed for MHC class I expression. The results indicated that in most CIN lesions only a minority of CTLs are activated, whereas in some carcinomas a massive infiltration of activated, i.e. granzyme B-positive, CTLs were observed. The percentage of activated CTLs was not related to expression of MHC class I on neoplastic cells. These results suggest that in some carcinomas proper activation of CTLs occurs but that most likely local factors or immunoselection of resistant neoplastic cells inhibit a proper response of CTLs to these neoplastic cells.

**Keywords:** Cervical carcinoma; cervical dysplasia; cytotoxic T lymphocyte; granzyme B; major histocompatibility complex

Molecular biological and epidemiological studies have shown that infection with certain human papillomavirus (HPV) genotypes is strongly associated with the development of cervical carcinoma (Zur Hausen, 1994; IARC, 1995). Follow-up studies of women with cytomorphological abnormal cervical smears indicate that persistence of oncogenic HPV types is conditional for the progression to high grade cervical intraepithelial neoplasia (CIN) III (Ho et al 1995; Remmink et al 1995). However, the vast majority of CIN lesions regress spontaneously. As in HPV-induced regressing warts, CIN lesions are infiltrated by T cells, indicating that there might be a local immune response against HPV (Coleman et al, 1994). A failure of the immune response against HPV might result in persistence of the infection and a subsequent progression of the lesion. An active role for the cellular immune system in the prevention of the development of cervical cancer is evidenced by a higher incidence of HPV-associated premalignant lesions in immunocompromised patients, such as AIDS patients and transplant recipients (Halpert et al, 1986; Laga et al, 1992). However, we and others have shown that major histocompatibility complex (MHC) class I expression is frequently down-regulated on neoplastic cells in malignant and premalignant lesions (Connor and Stern, 1990; Cromme et al, 1993; Honma et al, 1994; Keating et al, 1995). Lower expression or even complete absence of MHC class I molecules on tumour cells could affect their lysis by cytotoxic T cells as has been shown in melanomas (Rivoltini et al, 1995).

*Received 9 January 1997*
*Received 25 April 1997*
*Accepted 1 May 1997*

*Correspondence to:* JA Kummer

Cytotoxic T cells (CTL) and natural killer (NK) cells are the major effector cells in eradicating virus-infected cells. Activated $CD8^+$ CTL and NK cells hold cytotoxic granules that contain the lytic products perforin (Lichtenheld et al, 1988), granzymes: a group of highly homologous serine proteases (Jenne and Tschopp, 1988) and the T-cell-restricted intracellular antigen (TIA-1) (Tian et al, 1991). Perforin, or pore-forming protein, causes cell lysis by generating small pores in the plasma membrane of the target cell (Lichtenheld et al, 1988). Recently, the role of granzymes in target cell death has been extensively studied. Studies with granzyme B (GrB) knockout mice have shown that granzyme B plays a crucial role in CTL and NK-cell-mediated target cell apoptosis (Heusel et al, 1994).

Granzymes are specifically expressed by activated CTLs, as has been shown in vivo in autoimmune and infectious diseases, during allograft rejection and in lymphomas (Griffiths and Müller, 1991; Oudejans et al, 1996). Moreover, in vitro stimulation of lymphocytes with, for example, IL-2 or T-cell mitogens (MAb against CD3 combined with PMA) induces granzyme mRNA and protein expression (Liu et al, 1989). GrB is therefore a good marker for the identification of activated CTLs. TIA-1 is a recently identified granule-associated RNA-binding protein that may also play a role in target cell apoptosis (Tian et al, 1991). In contrast to GrB, TIA-1 is constitutively expressed in more than 50% of peripheral blood $CD8^+$ T cells and thus may define a subpopulation of $CD8^+$ T cells possessing cytolytic potential (Anderson, 1996). However, expression of TIA-1 is also strongly up-regulated after activation.

**Table 1** MHC class I and infiltrate analysis in cervical carcinoma

| CxCa number | HPV | Neoplastic cells | | Mean number of infiltrating cells per HPF | | | |
|---|---|---|---|---|---|---|---|
| | | HC-A2 HLA-A[a] | HC-10 HLA-B/C[a] | CD3 | CD8 | TIA-1 | GrB/CD3 |
| 1 | 18 | + | + | 55.5 | 21.4 | 11.0 | 22.3 |
| 2 | X | + | + | 44.2 | 23.3 | 13.8 | 6.9 |
| 3 | 16, 18 | + | + | 46.7 | 27.5 | 8.4 | 7.5 |
| 4 | X | + | + | 24.7 | 13.2 | 4.5 | 4.4 |
| 5 | 16 | + | + | 15.8 | 14.9 | 8.0 | 9.2 |
| 6 | 16 | + | ± | 8.0 | 3.6 | 5.1 | 1.6 |
| 7 | 18 | + | ± | 15.4 | ND | 8.2 | 6.0 |
| 8 | 16 | + | ± | 31.5 | 20.3 | 6.9 | 10.0 |
| 9 | 16 | ± | ± | 28.6 | 31.8 | 10.8 | 6.0 |
| 10 | 6, 16 | + | – | 46.3 | 48.4 | 18.5 | 44.7 |
| 11 | 16 | – | ± | 6.2 | 4.9 | 5.7 | 1.7 |
| 12 | 16 | – | – | 41.5 | 33.2 | 25.4 | 21.1 |
| 13 | 16 | – | – | 20.0 | 18.5 | 13.7 | 3.1 |
| 14 | 6, 16, 18 | – | – | 63.4 | 73.6 | 63.9 | 72.4 |

[a]+, Normal expression > 75% positive; ±, heterogeneous expression 25–75% strongly reduced to negative; –, disturbed expression > 75% strongly reduced to negative. ND, not done.

In the present study we have analysed the the number and activation state of T cells with a cytotoxic phenotype and NK cells as determined by CD3, CD8, TIA-1 and GrB expression in CIN lesions of increasing severity and cervical carcinoma. Furthermore, the presence of activated CTL and NK cells was related to MHC class I expression on neoplastic cells. In contrast to what was expected, the number of activated CTLs was increased in invasive carcinoma compared with CIN lesions. However, the presence of activated CTLs was not related to MHC class I expression on the atypical cells.

### MATERIALS AND METHODS

### Patients and tissues

Fourteen squamous cell carcinomas of the uterine cervix and 24 CIN lesions with different degrees of dysplasia (grade I, $n$ = 7; grade II, $n$ = 7; and grade III, $n$ = 10), randomly chosen from patients attending the oncological gynaecological outpatient department of the Free University Hospital, were analysed. The presence of HPV DNA was assessed by a general primer-based polymerase chain reaction (PCR) method, as previously described (Van den Brule et al, 1990). Positive samples were subjected to a type-specific PCR identifying the following HPV types: HPV 6, 11, 16, 18, 31 and 33. When HPV DNA was present, but not as one of the above-mentioned types, an X was assigned. Formalin-fixed paraffin embedded tissues were cut into 4-µm-thick sections and mounted on 3-amino-propyl-triethoxy-silane-coated slides (APES; Sigma, MO, USA) for haematoxylin and eosin (HE) and immunohistochemical staining.

### Immunohistochemistry

*Monostaining*

The expression of HLA-A [HC-A2, mouse monoclonal antibody (MAb), 1:500 (Stam et al, 1990)], HLA-B/C [mouse MAb HC-10, 1:1000 (Stam et al, 1990)], CD3 (rabbit PolyAb, Dakopatts, 1:500), CD8 (mouse IgG1 MAb, a generous gift from Dr Mason, Oxford, UK, 1:10), TIA-1 (mouse MAb, coulter clone, UK, 1:250) and granzyme B [mouse IgG2a MAb GrB7, 1:500; mouse IgG1 MAb GrB9 (Kummer et al, 1993, 1995)] were analysed as described previously (Sale et al, 1994; Cromme et al, 1995).

Briefly, formalin-fixed paraffin-embedded sections were deparaffinized using xylene; endogenous peroxidase was blocked and antigen retrieval by microwave treatment was performed for staining with the CD3, CD8, TIA-1 and GrB7 antibodies. Sections stained with HC-A2 were pretreated with target unmasking fluid (TUF, monosan, Uden, The Netherlands). Sections were subsequently washed and preincubated with normal serum and incubated with the primary antibody. Primary antibodies were detected using biotinylated secondary antibodies, followed by horseradish peroxidase (HRP)-conjugated streptavidin. HRP activity was detected by incubating the slides with diaminobenzidine (DAB) and hydrogen peroxide. The sections were counterstained with haematoxylin, dehydrated and mounted. As a control, sections were incubated with an irrelevant MAb of the appropriate subclass.

GrB9 and GrB7 showed a clear difference in specificity. GrB9 stained not only lymphocytes but also polymorphonuclear leucocytes, owing to cross-reaction with homologous serine proteases present in these cells (Kummer et al, 1995) and thereby hindering easy scoring. GrB7 did not show such cross-reactivity and showed a better sensitivity than GrB9. Therefore, the GrB7 monoclonal antibody was used in this study.

*Double staining*

Double staining was performed for GrB and CD3 as described previously (Oudejans et al, 1996). After boiling in a citrate buffer, sections were simultaneously incubated with both antibodies. After washing, GrB was detected using biotin-labelled goat anti-mouse IgG2a antibody and streptavidin-HRP. HRP was visualized by incubation for 10 min with 0.2 mg $ml^{-1}$ DAB, 0.002% hydrogen peroxide 0.07% nickel chloride in 50 mM Tris-HCl, pH 7.6, resulting in a DAB–nickel precipitate.

After blocking the remaining peroxidase activity with 0.3% hydrogen peroxide–methanol for 15 min, the slides were lightly fixed with 4% paraformaldehyde. CD3 was subsequently detected using biotin-labelled swine anti-rabbit antibody and the streptavidin–biotin horseradish peroxidase complex (ABC, Dako, Glostrup, Denmark) respectively. HRP was visualized using DAB

© Cancer Research Campaign 1997

**Figure 1** CIN II lesion (CIN 12; Table 2) stained for (**A**) granzyme B and (**B**) CD8. Only a few granzyme B-positive cells, indicated by the small arrows (**A**), are seen compared with the number of CD8-positive cells (**B**). (**C**) CIN II (CIN 13; Table 2) lesion stained for TIA-1. (**D**) CIN III (CIN23; Table 2) lesion stained for granzyme B. Size bars represent 30 μm and hydrogen peroxide, resulting in a clear brown signal for CD3. Subsequently, silver enhancement of the DAB–nickel precipitate was performed as described previously (Merchentaler et al, 1989), resulting in a black-staining signal for GrB. The sections were counterstained with haematoxylin, dehydrated and mounted.

### Interpretation of immunohistochemical staining

Immunohistochemical results were interpreted by three independent observers. Briefly, MHC class I expression was scored according to the percentage of neoplastic cells that showed staining. Reduced staining on neoplastic cells was determined compared with internal positive controls, such as normal squamous epithelium and lymphocytes. Lesions were scored as positive (+) when the majority of neoplastic cells (> 75%) showed membranous staining; as heterogeneous (±) when areas of positively stained neoplastic cells were observed next to negative areas (the latter constituting 25–75% of the neoplastic cells); as negative (–) when the majority of the neoplastic cells (> 75%) showed no membranous staining.

Infiltrate analysis of the CIN lesions was performed interpreting at least ten high-power fields (HPF, 400×) or, when the biopsy was small, the complete area of dysplastic epithelium. Where possible, each HPF consisted of at least 50% epithelial cells. In carcinomas ten HPFs were systematically randomly selected using an interactive video-overlay-based measuring system (Q-PRODIT, Leica,

© Cancer Research Campaign 1997

**Table 2** MHC class I and infiltrate analysis of the CIN lesions

| | | Neoplastic cells | | Mean number of infiltrating cells per HPF | | | |
|---|---|---|---|---|---|---|---|
| Grade | HPV | HC-A2 HLA-A[a] | HC-10 HLA-B/C[a] | CD3 | CD8 | TIA-1 | GrB |
| CIN I | | | | | | | |
| 1 | 18, X | + | + | 13.3 | 5.3 | 8.2 | 4.5 |
| 2 | X | + | + | 15.5 | 14.0 | 3.9 | 1.3 |
| 3 | Negative | + | + | 9.1 | 18.9 | ND | 1.6 |
| 4 | 6 | + | ± | 17.4 | 7.1 | 2.6 | 0.8 |
| 5 | Negative | + | ± | 12.2 | 11.8 | 5.3 | 5.6 |
| 6 | 6 | + | – | 14.8 | 5.4 | 12.6 | 1.0 |
| 7 | Negative | ± | – | 24.6 | 14.4 | 4.4 | 0.7 |
| CIN II | | | | | | | |
| 8 | 16 | + | + | 42.3 | 33.1 | 11.6 | 0.8 |
| 9 | 18, X | + | + | 23.8 | 19.0 | 5.2 | 1.6 |
| 10 | X | + | ± | 30.3 | 17.0 | 12.7 | 1.4 |
| 11 | 16 | + | ± | 28.6 | 23.4 | 1.8 | 0.7 |
| 12 | 16, 31 | ± | ± | 27.3 | 34.3 | ND | 3.0 |
| 13 | 33 | + | – | 24.6 | 14.3 | 5.3 | 2.2 |
| 14 | 16 | ± | – | 25.1 | 20.5 | 6.2 | 2.3 |
| CIN III | | | | | | | |
| 15 | 16 | + | + | 40.9 | 37.7 | 13.1 | 1.8 |
| 16 | 6, 16 | + | ± | 18.1 | 12.6 | 0.4 | 0.9 |
| 17 | X | + | ± | 8.5 | 7.1 | 5.3 | 1.7 |
| 18 | 16 | ± | ± | 21.6 | 17.3 | 8.6 | 2.6 |
| 19 | 16 | ± | ± | 38.1 | 21.7 | 7.9 | 2.4 |
| 20 | 31 | ± | ± | 17.1 | 13.3 | 6.8 | 2.4 |
| 21 | 33 | ± | – | 17.5 | 10.1 | 5.3 | 1.1 |
| 22 | 16 | ± | – | 8.7 | 2.6 | 2.4 | 0 |
| 23 | 33 | ± | – | 14.0 | 15.4 | 3.1 | 1.5 |
| 24 | 16 | ± | – | 13.7 | 11.1 | 3.8 | 0.3 |

[a]+, Normal expression > 75% positive; ±, heterogeneous expression 25–75% strongly reduced to negative; –, disturbed expression > 75% strongly reduced to negative. ND, not done.

Cambridge, UK). Infiltrating cells positive for CD3, CD8, TIA-1, GrB7 and CD3/GrB7 double-positive cells were counted by two independent observers.

### Statistical methods

The expression of the different markers in the different grades of dysplasia were compared using the Mann–Whitney two-sample test and the Wilcoxon signed rank test.

### RESULTS

### HPV DNA detection

Three CIN lesions, all CIN I, were HPV negative. The HPV types detected in the other CIN lesions were HPV 6, 16, 18, 31, 33 with the exception of four CIN lesions in which a type other than HPV 6, 11, 16, 18, 31 and 33 (designated X) was detected (Table 2). All carcinomas were HPV positive: in 12 cases a high-risk type was present (HPV 16 or 18) and in two carcinomas a HPV-X (Table 1).

### Granzyme B and TIA-1 activity in cervical intra-epithelial neoplasia

A few $GrB^+$ cells were observed in CIN lesions both in stroma and infiltrating in the neoplastic areas (Table 2). $GrB^+$ cells were frequently detected under the squamous–columnar junction. In the majority of the CIN lesions, $GrB^+$ lymphocytes were sporadically detected, in spite of substantial infiltration by $CD3^+CD8^+$ CTILs in the dysplastic area (Figure 1A and B, Table 2). In general, the staining intensity of the $GrB^+$ lymphocytes in CIN lesions was much weaker than that of $GrB^+$ cells in carcinomas. Not only were CTLs found in or under the dysplastic epithelium but also considerable numbers of CTLs were detected under the normal squamous epithelium. These cells were nearly all $GrB^-$ (not shown). The mean number of TIA-1-positive cells in CIN I–III was significantly higher than the mean number of $GrB^+$ cells (6.2 vs 1.7; $P = 0.0001$, Wilcoxon signed rank test; Fig. 1C and D, Table 2). There were no differences among the different grades of CIN. In two cases, there were more TIA-1-positive cells than CD8-positive cells, probably indicating the presence of CD3- and CD8-negative NK cells.

### Granzyme B and TIA-1 activity in carcinomas

The number of tumour-infiltrating, granzyme B-expressing, lymphocytes varied substantially between the different carcinomas, ranging from just a few lymphocytes per HPF (i.e. CxCa 2, 4, 6 and 13; Table 1) to a massive infiltration over 40 $GrB^+$ lymphocytes per HPF (i.e CxCa 10 and 14; Table 1 and Figure 2A). In the majority of the tumours, $GrB^+$ cells were located predominantly in the neoplastic area in close contact with the tumour cells (Figure 2A). In the stromal tissue, a marked intra- and perivascular localization of $GrB^+$ cells was observed (Figure 2D).

To determine whether granzyme B-expressing lymphocytes represented $CD3^+$ T lymphocytes or $CD3^-$ NK cells, double staining with GrB7 and CD3 was performed (Figure 2A and D). To

© Cancer Research Campaign 1997

**Figure 2** An HLA-A negative (**A–C**) and a HLA-A positive (**D–F**) carcinoma were stained for both CD3 (brown) and granzyme B (black) (**A** and **D**), CD8 (**B** and **E**) and for HLA-A (**C** and **F**). The stained cells in **C** are MHC class I-positive infitrating macrophages and lymphocyes, acting as an internal positive control. In **D** $GrB^+$ $CD3^+$ CTLs are indicated by the small arrows and a $GrB^+$ $CD3^-$ NK cell with a LGL morphology is indicated by a large arrow. Size bars represent 30 µm discriminate between $CD4^+$ T-helper lymphocytes and $CD8^+$ cytotoxic T lymphocytes, CD8 staining was performed on subsequent tissue sections (Figure 2B and E).

In general, the majority of the $GrB^+$ lymphocytes, infiltrating in the neoplastic area stained positive for CD3 (Figure 2A). CD8 staining of subsequent sections showed that the $CD3^+GrB^+$ cells

© Cancer Research Campaign 1997

**Figure 3** Mean CD8/CD3, TIA-1/CD3 and GrB/CD3 ratios of infiltrating cells in CIN lesions (CINI-III, ■) and in cervical carcinomas (▩). Significant differences are indicated by the asterisks also expressed CD8 (Figure 2A and B), indicating that these represent activated CTLs. $GrB^+$ but $CD3^-$ lymphocytes, representing NK cells, were observed in all tumours. In most tumours, they were a minor population compared with the number of $CD3^+$ $GrB^+$ lymphocytes (not shown). $GrB^+CD3^-$ NK cells showed a distinct morphology of large granular lymphocytes and showed an intense granzyme B staining (Figure 2D).

The mean number of TIA-1-positive cells was not significantly different from the mean number of $GrB^+$ cells (14.6 vs 14.9; $P$ = 0.36, Wilcoxon signed rank test; Table 1). The percentage of $CD8^+$ lymphocytes that are positive for granzyme B varies between the different tumours from approximately 15% in C × Ca 13, to up to 100% in CxCa 10 (Table 1). These results indicate that in most carcinomas the majority of the $CD8^+$ cells with cytotoxic potential are indeed activated. In contrast, only a minority of the CTL present in stroma (< 5%) showed an activated profile.

The ratio of infiltrating CD8/CD3 cells was similar in the CIN lesions and in the carcinomas. However, the mean TIA-1/CD3 ratio was slightly, though not significantly, higher in the carcinomas than in the CIN lesions (Figure 3, $P = 0.083$, Mann–Whitney two sample test). The GrB7/CD3 ratio was significantly higher in the carcinomas than in the CIN lesions (Figure 3; $P < 0.0001$, Mann–Whitney two-sample test).

### MHC class I expression on neoplastic cells

All CIN lesions were positive for HLA-A and/or HLA-B/C: three (43%) CIN I; five (72%) CIN II; and nine (82%) CIN III lesions showed heterogeneous expression of MHC class I or a complete loss for HLA-B/C alone (Table 2).

In 5 out of 14 carcinomas (35%), normal HLA-A and HLA-B/C expression was observed and six (43%) showed heterogeneous expression. Three (21%) carcinomas were completely negative for MHC class I (Table 1).

As CTL killing is usually MHC class I restricted, infiltration of $GrB^+$ cells was related to HLA-A and HLA-B/C expression. No correlation between HLA class I expression and the number of granzyme B-expressing cells was found. Numerous infiltrating $GrB^+$ CTLs can be observed in both MHC class I-negative tumours (Figure 2A–C) as well as in tumours with a normal MHC class I expression (Figure 2D–F). The number of $CD8^+$ cells alone did not correlate with MHC class I expression either.

### DISCUSSION

In this study the question was assessed whether there is a role for cytotoxic T cells in (pre)malignant lesions of the cervix. We found that the percentage of activated $CD3^+$ cytotoxic T cells as demonstrated by CD8, TIA-1 and GrB expression was higher in invasive carcinoma than in premalignant CIN I–III. The fact that in a previous study (Cromme et al, 1995) fewer activated CTLs were detected is due to the antibody used, i.e. GrB9, which is less specific and sensitive than the GrB7 MAb used in this study.

Interestingly, most CIN lesions were infiltrated by a substantial number of $CD8^+$ lymphocytes in the dysplastic epithelium of which approximately 50% expressed TIA-1 (Figure 3), but $GrB^+$ lymphocytes were only sporadically present. These results indicate that in CIN only a minority of infiltrating $CD8^+$ lymphocytes with cytotoxic potential are indeed activated. No clear relation could be demonstrated between MHC class I expression and the number of activated CTLs infiltrating in the epithelium.

In most carcinomas numerous GrB-infiltrating lymphocytes were present. The number varied substantially, ranging from just a few lymphocytes per HPF to a massive infiltration up to 70 $GrB^+$ lymphocytes per HPF. Phenotyping showed that the majority of $GrB^+$ lymphocytes were $CD8^+$ CTL (Figure 2), although, in some, a substantial number of NK cells were present (data not shown). The percentage of $CD8^+$ lymphocytes that were positive for GrB varied between the different tumours from approximately 15% up to 100%. The number of TIA-1-expressing cells was not significantly different from the number of GrB-expressing cells, indicating that in carcinomas the majority of CTLs with cytotoxic phenotype (TIA-1 positive) are indeed activated ($GrB^+$). However, in some carcinomas only a minority of $CD8^+$ T cells expressed GrB and/or TIA-1, indicating that in these carcinomas a lack of CTL activation is observed. Thus, compared with CIN, in carcinomas more $CD8^+$ cells with cytotoxic potential and significantly more activated CTLs were found infiltrating in the epithelium (Figure 3). In the stromal tissue, a marked intra- and perivascular localization was observed (Figure 2D), indicating that a proportion of the cytotoxic lymphocytes were activated before or during infiltration of the tumour.

Most CIN lesions showed poor infiltration of $GrB^+$ cells in the dysplastic epithelium. The question arises whether these few activated CTLs upon recognition of the virus-infected keratinocytes, are sufficient to induce regression of these lesions. A single CTL is capable of killing many different targets one after another, and a recent study has shown that the granzyme–perforin granule pathway can be sustained during T-cell killing (Isaaz et al, 1995). Although the relatively low number of activated CTLs suggest that CTLs do not play an important role in the spontaneous regression of CIN lesions, it cannot be ruled out that a limited number of activated CTLs can kill a sufficient number of keratinocytes in these lesions to initiate regression.

In addition, the presence of large number of activated CTLs detected in some of the cervical carcinomas is not sufficient to eradicate the tumour. Several explanations for this observation are possible. First, it could be simply that there are still too few CTLs and that the neoplastic cells proliferate faster than CTLs can kill them.

Secondly, MHC class I alleles are down-regulated on the surface of neoplastic cells, as has been previously shown in several studies for both premalignant and malignant lesions (Connor and Stern, 1990; Cromme et al, 1993). This lower expression or even complete absence of MHC class I molecules on tumour cells could

© Cancer Research Campaign 1997

affect their recognition and lysis by CTLs (Rivoltini et al, 1995). In agreement with the above-mentioned studies, most carcinomas showed (partial) down-regulation of MHC class I alleles, but no relation between the presence of activated CTLs and expression of MHC class I in the carcinomas tested was observed. High numbers of $GrB^{+}CD3^{+}$ CTLs were observed in contact with keratinocytes that were completely negative for MHC class I (Figure 2A–C) as well as in neoplastic areas expressing normal levels of MHC class I (Figure 2D–F). Although studies on the effector mechanisms of NK cells indicate that a down-regulation of particular MHC class I alleles makes the neoplastic cells more sensitive to NK-cell-mediated lysis (Lanier and Phillips, 1996), NK cells were not found in higher frequencies in MHC class I down-regulated neoplastic areas (not shown). Most probably, in those carcinomas in which there is a massive activated CTL infiltrate but a lack of MHC class I expression on the neoplastic cells, immunoselection by CTLs of resistant tumour cells has taken place. A similar mechanism has been suggested in Epstein–Barr virus (EBV)-associated Hodgkin's disease (Poppema and Visser, 1994). Alternatively, CTLs may detect lower MHC class I levels than are detected by the antibodies used in this study. Furthermore, shedding of MHC class I or shed tumour antigen–antibody complexes has been postulated to be a blocking factor that can interfere with the immune response (Giacomini et al, 1984).

Thirdly, resistance of tumour cells can occur owing to other factors than those related to MHC class I, for example CTL-induced apoptosis is dependent on the state of activation of the target cell and its commitment into the mitotic cycle (Nishioka and Welsh, 1994) and target cell mutants have been described that are recognized by, and provide an antigenic stimulus to, the CTLs, whereas they have lost the capacity to respond by dying (Ucker et al, 1995). Also an, at present unknown, protective membrane protein that impairs perforin-mediated cytolysis has been described for lymphoid cells (Muller and Tschopp, 1994), but it is still not known whether other cell types possess such protective molecules. Recently, it was shown that the expression of bcl-2, a marker for inhibition of programmed cell death, increased with the severity of cervical (pre)malignant lesions. These results indicate that bcl-2 might play a role in rendering transformed keratinocytes resistant to apoptotic cell death (ter Harmsel et al, 1996).

Finally, the observed massive $GrB^{+}$ T-cell infiltrate might be part of a general inflammatory reaction, due to local cytokine production, rather than a consequence of a specific immune response. Although synthesis of lytic proteins and the formation of lytic granules is triggered by recognition of a target cell by the T-cell receptor of the CTL precursor (Isaaz et al, 1995), non-specific activation, for example by interleukin 2, is possible (Kummer et al, 1995). Interleukin 2 production in these tumours is currently under investigation.

Some carcinomas in this study have low numbers of activated CTLs, in spite of a substantial $CD8^{+}$ T-cell infiltrate. T-helper type 1 (Th1) cells produce cytokines principally providing help for CTL-mediated responses, whereas Th2 cells provide help mainly for antibody-mediated responses. Cytokines, produced, for example, by the tumour cells, such as interleukin 10 and transforming growth factor-β (TGF-β), can shift the local balance towards a more Th2-type response or directly inhibit CTL activation and thus explain a lack of infiltrating functional activated CTLs as was previously suggested for EBV-specific CTLs in EBV-positive Hodgkin's disease (Frisan et al, 1995; Oudejans et al, 1996). We are currently investigating this hypothesis. Preliminary RT PCR results show that local production of the immunosuppressive cytokine TGF-β (Xie and Gallagher, 1994) might be responsible for a lack of CTL activation in these carcinomas. Further in vitro studies are necessary to substantiate this observation.

In the mouse, it has been shown for cytopathic viruses [vesicular stomatitis virus (VSV) and semliki forest virus (SFV)] that soluble mediators play a role in eradication of the virus rather than cell-mediated factors, although the cell-mediated immune response plays an important role in the eradication of non-cytopathic viruses such as lymphocytic choriomeningitis virus (LCMV) (Kägi et al, 1995). The lack of CTL activation in CIN compared with most carcinomas might be due to these mechanisms. In low-grade CIN, HPV infection is thought to be a productive lytic infection, possibly because it does not allow enough time for the infected cell to process and present HPV antigens. Thus, at this stage, non-lytic T-cell-dependent soluble mediators such as cytokines (interferon-γ) and neutralizing antibodies might play a more important role. As HPV infection is non-cytopathic in carcinomas, allowing processing and presentation of HPV antigens, cell-mediated factors might play a more important role at this stage of the disease.

In conclusion, our results show that there is a distinct infiltration by $CD8^{+}$ T-lymphocytes in both CIN lesions and carcinoma of the cervix, but surprisingly the percentage of activated T-cells, as measured by GrB expression, increases from CIN lesions to carcinomas. These results indicate that in certain cervical carcinomas, local factors, presumably cytokines or immunoselection of the tumour cells, are responsible for escaping the immune response, rather than a lack of CTL activation a priori. The role of these local factors is currently under investigation in our laboratory.

### ACKNOWLEDGEMENT

The authors would like to thank H. F. J. Schrijnemakers for his excellent technical assistance.

### REFERENCES

Anderson P (1996) TIA-1: Structural and functional studies on a new class of cytotoxic effector molecule. *Curr Topics Microbiol Immunol* **198**: 131–143

Coleman N, Birley HDL, Renton AM, Hanna NF, Ryait BK, Byrne M, Taylorrobinson D and Stanley MA (1994) Immunological events in regressing genital warts. *Am J Clin Pathol* **102**: 768–774

Connor ME and Stern PL (1990) Loss of MHC class-I expression in cervical carcinoma. *Int J Cancer* **46**: 1029–1034

Cromme FV, Meijer CJLM, Snijders PJF, Uyterlinde A, Kenemans P, Helmerhorst T, Stern PL, Van den Brule AJC and Walboomers JMM (1993) Analysis of MHC class-I and class-II expression in relation to presence of HPV genotypes in premalignant and malignant cervical lesions. *Br J Cancer* **67**: 1372–1380

Cromme FV, Walboomers JMM, Stukart MJ, de Gruijl TD, Kummer JA, Leonhart AM, Helmerhorst TJM and Meijer CJLM (1995) Lack of granzyme expression in T lymphocytes indicates poor cytotoxic T lymphocyte activation in human papillomavirus-associated cervical carcinomas. *Int J Gynecol Cancer* **5**: 366–373

Frisan T, Sjoberg J, Dolcetti R, Boiocchi M, De Re V, Carbone A, Brautbar C, Battat C, Biberfeld P, Eckman M, Ost A, Christensson B, Sundstrom C, Bjorkholm M, Pisa P and Masucci MG (1995) Local suppression of Epstein Barr virus (EBV)-specific cytotoxicity in biopsies of EBV-positive Hodgkin's disease. *Blood* **86**: 1493–1501

Giacomini P, Aguzzi A, Pestka S, Fisher PB and Ferrone S (1984) Modulation by recombinant DNA leukocyte (alpha) and fibroblast (beta) interferons of the expression and shedding of HLA- and tumor-associated antigens by human melanoma cells. *J Immunol* **133**: 1649–1655

Griffihs GM and Müller C (1991) Expression of perforin and granzymes in vivo: potential diagnostic markers for activated cytotoxic cells. *Immunol Today* **12**: 415–119

© Cancer Research Campaign 1997

Halpert R, Fruchter RG, Sedlis A, Butt K, Boyce JG and Sillman FH (1986) Human papillomavirus and lower genital neoplasia in renal transplant patients. *Obstet Gynecol* **68**: 251–258

Heusel JW, Wesselschmidt RL, Shresta S, Russell JH and Ley TL (1994) Cytotoxic lymphocytes require granzymes B for the rapid induction of DNA fragmentation and apoptosis in allogeneic target cells. *Cell* **76**: 977–987

Ho GYF, Burk RD, Klein S, Kadish AS, Chang CJ, Palan P, Basu J, Tachezy R, Lewis R and Romney S (1995) Persistent genital human papillomavirus infection as a risk factor for persistent cervical dysplasia. *J Natl Cancer Inst* **87**: 1365–1371

Honma S, Tsukada S, Honda S, Nakamura M, Takakuwa K, Maruhashi T, Kodama S, Kanazawa K, Takahashi T and Tanaka K (1994) Biological-clinical significance of selective loss of HLA-class-I allelic product expression in squamous-cell carcinoma of the uterine cervix. *Int J Cancer* **57**: 650–655

IARC (1995) The Human Papillomavirus, *Monographs on the Evaluation of the Carcinogenic Risks to Humans*, vol. 64, International Agency for Research on Cancer: Lyon

Isaaz S, Baetz K, Olsen K, Podack E and Griffiths GM (1995) Serial killing by cytotoxic T lymphocyes: T cell receptor triggers degranulation, re-filling of the lytic granules and secretion of lytic proteins via a non-granule pathway. *Eur J Immunol* **25**: 1071–1079

Jenne DE and Tschopp J (1988) Granzymes, a family of serine proteases released from granules of cytolytic T lymphocytes upon T cell receptor stimulation. *Immunol Rev* **103**: 53–71

Kägi D, Seiler P, Pavlovic J, Lederman B, Zinkernagel RM and Hengartner H (1995) The roles of perforin- and Fas-dependent cytotoxicity in protection against cytopathic and noncytopathic viruses. *Eur J Immunol* **25**: 3256–3262

Keating PJ, Cromme FV, Duggan-Keen M, Snijders PJF, Walboomers JMM, Hunter RD, Dyer PA and Stern PL (1995) Frequency of down regulation of individual HLA-A and -B alleles in cervical carcinomas in relation to TAP-1 expression. *Br J Cancer* **72**: 405–411

Kummer JA, Kamp AM, van Katwijk M, Brakenhoff JPJ, Radosevic K, van Leeuwen AM, Borst J, Verweij CL and Hack CE (1993) Production and characterization of monoclonal and polyclonal antibodies raised against recombinant human granzymes A and B and cross reacting with the natural proteins. *J Immunol Methods* **163**: 77–83

Kummer JA, Kamp AM, Tadema TM, Vos W, Meijer CJLM and Hack CE (1995) Localization and identification of granzymes A and B-expressing cells in normal human lymphoid tissue and peripheral blood. *Clin Exp Immunol* **100**: 164–172

Laga M, Icenogle JP, Marsella R, Manoka AT, Nzila N, Rynder RW, Vermund SH, Heyward WL, Nelson A and Reeve WC (1992) Genital papillomavirus infection and cervical dysplasia – opportunistic complications of HIV infection. *Int J Cancer* **48**: 682–688

Lanier LL and Phillips JH (1966) Inhibitory MHC class I receptors on NK cells and T-cells. *Immunol Today* **17**: 86–100

Lichtenheld MG, Olsen KL, Lu P, Lowrey DM, Hameed A, Hengartner H and Podack ER (1988) Structure and function of human perforin. *Nature* 448–451

Liu CC, Rafii S, Granelli-Piperno A, Trapani JA and Young J-E (1989) Perforin and serine esterase gene expression in stimulated human T cells. Kinetics, mitogen requirements, and effects of cyclosporin A. *J Exp Med* **170**: 2105–2118

Merchentaler I, Stankovics I and Gallyas F (1989) A highly sensitive one-step method for silver intensification of the nickle-diaminobenzidine end product of peroxidase reaction. *J Histochem Cytochem* **37**: 1563–1565

Muller C and Tschopp J (1994) Resistance of CTL to perforin-mediated lysis. Evidence for a lymphocyte membrane protein interacting with perforin. *J Immunol* **153**: 2470–2478

Nishioka WK and Welsh RM (1994) Susceptibility to cytotoxic T lymphocyte-induced apoptosis is a function of the proliferative status of the target. *J Exp Med* **179**: 769–774

Oudejans JJ, Jiwa NM, Kummer JA, Horstman A, Vos W, Baak JPA, Kluin PM, van der Valk P, Walboomers JMM and Meijer CJLM (1996) Analysis of major histocompatibility complex class I expression on Reed–Sternberg cells in relation to the cytotocix T-cell response in Epstein–Barr virus positive and negative Hodgkin's disease. *Blood* **87**: 3844–3851

Poppema S and Visser L (1994) Absence of HLA class I expression by Reed–Sternberg cells. *Am J Pathol* **145**: 37–41

Remmink AJ, Walboomers JMM, Helmerhorst TJM, Voorhorst FJ, Rozendaal L, Risse EKJ, Meijer CJLM and Kenemans P (1995) The presence of persistent high-risk HPV genotypes in dysplastic cervical lesions is associated with progressive disease: natural history up to 36 months. *Int J Cancer* **61**: 306–311

Rivoltini L, Barracchini KC, Viggiano V, Kawakami Y, Smith A, Mixon A, Restifo NP, Topalian SL, Simonis TB, Rosenberg SA and Marincola FM (1995) Quantitative correlation between HLA class I allele expression and recognition of melanoma cells by antigen-specific cytotoxic T lymphocytes. *Cancer Res* **55**: 3149–3157

Sale GE, Beauchamp L and Myerson D (1994) Immunohistologic staining of cytotoxic T and NK cells in formalin-fixed paraffin embedded tissue using microwave TIA-1 antigen retrieval. *Transplantation* **57**: 287–289

Stam NJ, Uroom TM, Peters PJ, Pastoors EB and Ploegh HL (1990) HLA-A and HLA-B specific monoclonal antibodies reactive with free heavy chains in western blots, in formalin fixed paraffin-embedded tissue sections and cryo-immuno-electron microscopy. *Int Immunol* **3**: 113–125

ter Harmsel B, Smedts F, Kuijpers J, Jeunink M, Trimbos B and Ramaekers F (1996) Bcl-2 immunoreactivity increases with severity of CIN: A study of normal cervical epithelia, CIN, and cervical carcinoma. *J Pathol* **179**: 26–30

Tian Q, Streuli M, Saito H, Schlossman SF and Anderson P (1991) A polyadenylate binding proteins localized to the granules of cytolytic lymphocytes induces DNA fragmentation in target cells. *Cell* **67**: 629–639

Ucker DS, Wilson JD and Hebshi LD (1995) Target cell death triggered by cytotoxic lymphocytes: a target cell mutant distinguishes passive pore formation and active cell suicide mechanism. *Eur J Immunol* **25**: 1163–1167

Van den Brule AJC, Meijer CJLM, Bakels V, Kenemans P and Walboomers JMM (1990) Rapid detection of human papillomavirus in cervical scrapes by combined general primer-mediated and type-specific polymerase chain reaction. *J Clin Microbiol* **28**: 2739–2743

Xie JW and Gallagher G (1994) The ability of transforming growth factor-beta 1 to preferentially inhibit the induction of cytotoxicity in human T cells is determined by the nature of the activating signals. *Anticancer Res* **14**: 1595–1598

Zur Hausen H (1994) Molecular pathogenesis of cancer of the cervix and its causation by specific human papillomavirus types. In *Human Pathogenic Papillomaviruses*, pp. 131–156. Springer: Berlin

© Cancer Research Campaign 1997